# Desigualdades raciais e de gênero em saúde na América Latina: tramas históricas e desafios cotidianos das mulheres negras

**DOI:** 10.1590/0102-311XPT227625

**Published:** 2026-08-31

**Authors:** Roberta Gondim de Oliveira, Ana Cláudia Barbosa

**Affiliations:** 1 Escola Nacional de Saúde Pública Sergio Arouca, Fundação Oswaldo Cruz, Fiocruz, Rio de Janeiro, Brasil.; 2 Instituto Federal de Educação, Ciência e Tecnologia do Rio de Janeiro, Rio de Janeiro, Brasil.

**Keywords:** Decolonialidade, Racismo, Interseccionalidade, Desigualdade em Saúde, América Latina, Decoloniality, Racism, Intersectionality, Health Status Dispararities, Latin America, Decolonialidad, Racismo, Interseccionalidad, Desigualdad en Salud, América Latina

## Abstract

O ensaio analisa as desigualdades sociais e de saúde que afetam mulheres latino-americanas, com foco nas mulheres negras, situando-as nas bases históricas e estruturais da colonialidade do poder, do saber e do ser. Objetiva problematizar como o racismo, o sexismo e o patriarcado - legados da modernidade colonial - conformam a determinação interseccional de iniquidades em saúde, sustentadas por sistemas políticos, econômicos e simbólicos que naturalizam a subalternização dos corpos racializados. Metodologicamente, trata-se de um ensaio teórico-analítico que articula literatura crítica, com base em referenciais decoloniais e interseccionais, e análise de documentos como relatórios e estudos regionais latino-americanos. O percurso argumentativo parte da conceituação de colonialidade em diálogo com autoras do feminismo negro, no sentido de evidenciar como a racialização, a classe e o gênero estruturam a distribuição desigual de oportunidades, riscos e mortes. O texto também examina alguns dados recentes, pré e pós-pandemia de COVID-19, sobre trabalho, violência, pobreza e saúde, destacando as persistentes assimetrias raciais e de gênero, indicando que as desigualdades interseccionadas se expressam de forma contínua e multifacetada nos campos econômico, social e sanitário, perpetuando hierarquias coloniais. Conclui-se que enfrentá-las requer políticas reparadoras e de justiça social com enfoque interseccional e decolonial, fortalecendo a autonomia das mulheres racializadas e a equidade nas políticas públicas de saúde.

## Abstract (en)

This study analyzes the social and health inequalities that affect Latin American women, situating Black women in the historical and structural bases of the coloniality of power, knowledge, and being. It aims to problematize how racism, sexism, and patriarchy − legacies of colonial modernity − shape the intersectional determination of health inequities sustained by political, economic, and symbolic systems that naturalize the subordination of racialized bodies. This theoretical-analytical essay articulates the critical literature based on decolonial and intersectional references and analyzes documents such as Latin American reports and regional studies. Its argumentative path conceptualizes coloniality in dialogue with Black feminism authors, evincing how racialization, class, and gender structure the unequal distribution of opportunities, risks, and deaths. It also examines some recent data (pre- and post-COVID-19 pandemic) on work, violence, poverty, and health, highlighting persistent racial and gender asymmetries that indicate continuous and multifaceted intersecting inequalities in the economic, social, and health fields and perpetuate colonial hierarchies. Addressing them requires intersectional and decolonial reparative and social justice policies, strengthening the autonomy of racialized women and equity in public health policies.

## Abstract (es)

Este ensayo analiza las desigualdades sociales y sanitarias que afectan a las mujeres latinoamericanas, centrándose en las mujeres negras y situándolas en los fundamentos históricos y estructurales de la colonialidad del poder, del saber y del ser. El objetivo es problematizar cómo el racismo, el sexismo y el patriarcado −legados de la modernidad colonial− configuran la determinación interseccional de las iniquidades en salud, sostenidas por sistemas políticos, económicos y simbólicos que naturalizan la subalternización de los cuerpos racializados. Metodológicamente, se trata de un ensayo teórico-analítico que articula la literatura crítica, basada en marcos decoloniales e interseccionales, y el análisis de documentos como informes y estudios regionales latinoamericanos. El recorrido argumentativo toma como punto de partida la conceptualización de la colonialidad en diálogo con autoras del feminismo negro, con el fin de destacar cómo la racialización, la clase y el género estructuran la distribución desigual de oportunidades, riesgos y muertes. El texto también examina algunos datos recientes, tanto anteriores como posteriores a la pandemia de COVID-19, sobre trabajo, violencia, pobreza y salud, destacando las persistentes asimetrías raciales y de género, lo que indica que las desigualdades inteseccionadas se expresan de forma continua y multifacética en los ámbitos económico, social y sanitario, perpetuando las jerarquías coloniales. Se concluye que para abordar estas cuestiones se requieren políticas reparadoras y de justicia social con un enfoque interseccional y decolonial, que fortalezcan la autonomía de las mujeres racializadas y la equidad en las políticas públicas de salud.

## Introdução

O contexto histórico social latino-americano marcado pelo colonialismo, como processo de dominação e exploração, e a colonialidade, racionalidade político-econômico-social e sua institucionalidade nas estruturas de poder, somados ao racismo escravista e ao patriarcado, compõem as bases da destituição de humanidade de povos racializados e da produção de desigualdades latino-americanas que recaem seletivamente sobre determinados grupos. Tendo por linha de base o contexto latino-americano, este ensaio debate as desvantagens sociais e de saúde que afetam mulheres latino-americanas de modo geral, enfocando mais detalhadamente as desigualdades que recaem sobre mulheres negras no contexto brasileiro, problematizando suas condições sociais historicamente forjadas e discutindo, em perspectiva interseccional, as iniquidades persistentes na região e a agência dessas mulheres na criação de alternativas ao sistema que as subalterniza.

A colonialidade pode ser entendida como o conjunto de expressões e efeitos da colonização, sustentada na exploração dos territórios e da força de trabalho de sujeitos racializados. A ausência de políticas de reparação histórica, após séculos de escravização e genocídio de povos africanos e indígenas, somada a matrizes de dominação como o patriarcado, recai desigualmente sobre grupos populacionais, especialmente sobre mulheres negras. O capitalismo expressa essa lógica por meio da violência em múltiplas formas − exercício da colonialidade do ser, do poder, do saber e de gênero ^1^ ^,^ ^2^ ^,^ ^3^. O Estado, em sua ação ou inação, representa uma das faces mais perversas desse processo, ao naturalizar social e subjetivamente as desigualdades.

O conceito da colonialidade é central na obra de Walter Mignolo ^3^, sendo entendida como o lado oculto da modernidade. Mignolo argumenta que a decolonialidade não deve ser um conceito totalitário, mas sim uma ideia que revela a modernidade e seu aspecto constitutivo: a colonialidade. Essa surge com as invasões europeias, a formação das Américas, do Caribe e o tráfico maciço de africanos escravizados, criando a Matriz Colonial de Poder. Mignolo também explora outros cenários, como colonização do tempo, com a criação de eras anteriores à colonização (como a Idade Média), e do espaço, com a conquista do “Novo Mundo” e novos marcos geográficos.

Baseando-se na Matriz Colonial de Poder, Mignolo ^3^ mostra que ela foi construída por nós histórico-estruturais heterogêneos que se interseccionam, dentre eles: (1) a questão da classe global, com a divisão internacional do trabalho entre centro e periferia; (2) a hierarquia racial/étnica, que favorecia os europeus sobre os não europeus; (3) a hierarquia de gênero e sexo, que privilegiava homens e criou a heteronormatividade; (4) a hierarquia epistêmica, que privilegiava o conhecimento ocidental; e (5) a ideia de sujeito moderno, centrada no homem branco heterossexual europeu, que foi a base para a classificação racial e o racismo global ^3^.

Em territórios marcados por contexto histórico colonial como o latino-americano, e em especial o brasileiro, as expressões da colonialidade se manifestam de modo particular nas relações raciais e de gênero. O cisheteropatriarcado branco impacta profundamente as condições de vida e saúde da população, perpetuando desigualdades históricas relativas entre outras à pobreza, à fome, às violências e à educação, conforme apresentado e debatido ao longo deste trabalho. Para Borret ^4^ (p. 3970), “*num país que se organiza socialmente a partir do colonialismo, do genocídio de povos nativos indígenas e de séculos de escravização e posterior marginalização de pessoas negras africanas*”, não é possível manter silêncio sobre o fato de que o racismo permeia os processos de saúde/adoecimento e, particularmente, o quanto ele “*se expressa nas interações terapêuticas e práticas de cuidado em saúde*” ^4^ (p. 3970).

A esse arranjo geopolítico histórico, ordenado com base na noção supremacista da branquitude, Bento ^5^ identifica ser inscrita como um sistema de dominação, estabelecendo-se como norma que provê sustento a privilégios em diversas esferas, incluindo a social e a da saúde. Ao posicionar a branquitude, em especial o homem branco europeu, como referência universal do “ser”, reforça-se que a modernidade se define por essa noção hierárquica de sujeitos de mundo. Essa lógica racializada determina o débil grau de cidadania e pertencimento ao humano concedido aos “não brancos”, frequentemente reduzidos à condição de “não pessoas” ^6^. Somado ao patriarcalismo, entendida nos moldes da colonialidade de gênero, destina à mulher “racializada” ainda mais subalternidade ^2^.

O texto percorre, portanto, três movimentos: revisita os fundamentos da interseccionalidade, compreendendo que a tríade gênero/raça/território opera desigualdades; analisa as expressões contemporâneas destas desigualdades no contexto da América Latina, corporificadas em mulheres latino-americanas como um todo, enfocando sobretudo e mais detidamente a realidade de mulheres negras brasileiras, baseando-se em discussões e análises ancoradas em dados recentes, pré e pós- pandemia de COVID-19, abordando dimensões como trabalho, violência, pobreza e saúde. Por fim, conclui destacando as potências políticas e epistemológicas dessas mulheres no agenciamento de novos horizontes de justiça e reexistência.

## Interseccionando as desigualdades

A formulação da interseccionalidade como conceito e ferramenta parte do reconhecimento de que certos grupos enfrentam desvantagens sociais e outros desfrutam de privilégios. Embora sua presença seja mais visível nos movimentos por justiça social do feminismo negro anglo-saxão a partir da década de 1970, suas sementes remontam às lutas das mulheres abolicionistas no século XIX ^7^. Um marco anterior ao termo é o Manifesto do Coletivo Combahee River, de 1977. Barbara Smith, uma das autoras, citada por Collins & Bilge ^8^ (p. 94) descreve esse Coletivo como “*um lugar sem homofobia, racismo ou sexismo*”, onde ela podia ser plenamente quem era − uma mulher negra e lésbica − sem deixar de lado nenhuma de suas identidades.

Ao transitar entre as ciências sociais e humanas, a noção interseccional de opressões vai além de descrever um fenômeno social: busca explicá-lo. Investiga o significado da condição humana, reconhecendo que o espaço social é mais do que a soma de indivíduos. “*A interseccionalidade é um conjunto de ideias críticas*” ^9^ (p. 79) sobre esse contexto, buscando nas narrativas e histórias de vida o sentido de estar no mundo.

O conceito foi sistematizado pela jurista e feminista Kimberlé W. Crenshaw em 1989, ganhando projeção internacional após a Conferência Mundial contra o Racismo, realizada em 2001, em Durban, África do Sul ^10^. A Declaração da conferência destacou as desvantagens associadas a gênero e etnicidade, instando os Estados a incorporar essa perspectiva em políticas públicas e ações de prevenção, educação e proteção contra múltiplas formas de discriminação, especialmente em contextos de pobreza e violência. Collins & Bilge ^8^ apontam duas questões centrais para o uso do conceito neste ensaio.

### A interseccionalidade-conceito

A primeira questão é que, embora Crenshaw tenha dado nome à ideia, permitindo seu trânsito interdisciplinar na academia, reconhecimento científico e legitimidade, a ideia não surgiu com ela - apesar dessa atribuição ser comum. Antes mesmo de Crenshaw nomear o conceito em 1989, Lélia Gonzalez ^11^, em *Racismo e Sexismo na Cultura Brasileira*, já discutia as matrizes de opressão que recaíam sobre as mulheres negras.

Entretanto, Crenshaw possuía as credenciais educacionais necessárias para atuar como tradutora confiável entre diferentes saberes. Além de nomear a interseccionalidade, ela inseriu em seus trabalhos perspectivas antirracistas, feministas e decoloniais, promovendo uma análise crítica que sustenta a resistência intelectual das pessoas historicamente oprimidas. Ao ser nomeada, a interseccionalidade se torna um conhecimento resistente, ligado ao ativismo e que valoriza “*a autorreflexão, a experiência e a ação social criativa como parte da busca por justiça social*” ^8^ (p. 180).

Carla Akotirene ^12^ afirma que o conceito de interseccionalidade foi gestado na mulher negra e resgata a célebre pergunta de Sojourner Truth, pioneira do feminismo negro: *“E Eu, Não Sou Uma Mulher?”*. Henning ^7^ também a destaca, ressaltando a complexidade dessa posição. Para Akotirene, racismo, capitalismo e cisheteropatriarcado são estruturas inseparáveis, comparadas a avenidas cujas interseções são cruzamentos onde se chocam gênero, raça e classe. O foco da interseccionalidade deve ser a “colisão no cruzamento”, evitando a reificação das categorias. Seu uso, segundo a autora, possibilita um ativismo teórico não exclusivista, atento à matriz colonial moderna e às múltiplas formas de interação entre as opressões.

Mesmo antes do termo interseccionalidade ser consagrado já se falava sobre a experiência das mulheres negras como Outras: Outras por não serem homens, Outras por não serem brancas. Segundo Akotirene ^12^, a análise dessa experiência não deve priorizar o corpo − o que ela considera o maior recurso colonial da eurocivilização − mas a humanidade, já que as mulheres são afetadas pelas iniquidades de gênero em intensidade e frequência distintas.

A interseccionalidade é simultaneamente uma teoria, uma metodologia e um instrumento prático, não se limitando à mera soma de conceitos ou a abordagens hierárquicas e comparativas de natureza matemática. Focada na experiência, a identidade não pode ser completa sem considerar todas as suas marcas, e a interseccionalidade promove um pensamento complexo e criativo, que não hierarquiza o sofrimento, mas busca soluções políticas para enfrentar a matriz de opressão que cria o Outro, o diferente, e reforça os privilégios da branquitude, perpetuando e reestruturando redes de desigualdade.

Como enfatiza Akotirene ^12^ (p. 48), “*a interseccionalidade trata da identidade atravessada pelo racismo e outras estruturas*”, reafirmando o protagonismo do racismo como eixo estruturante das demais opressões. A autora argumenta que a importância do termo está em destacar as diferenças, que são sempre relacionais (entre o sujeito que É e seu Outro), e em entender onde o racismo se soma às demais opressões, contextualizando-as.

Ao destaque fornecido ao racismo assinalamos ser este “*uma estrutura de dominação documentada há pelo menos quatro mil anos*” ^12^ (p. 79). Como resultado, opressões que poderiam ser ressignificadas, dependendo do contexto, permanecem e continuam a ser vividas de forma ainda mais cruel. Por isso, a interseccionalidade eleva sua crítica tanto ao movimento negro, que em sua origem reproduzia dinâmicas patriarcais, secundarizando questões de gênero, quanto ao marxismo, que privilegia a classe em detrimento de outras lutas que abordam as diversas tecnologias de poder ^13^.

Dedicada ao estudo do racismo, Grada Kilomba ^14^ (p. 65) aponta: “*No racismo estão presentes, de modo simultâneo, três características: a primeira é a construção de/da diferença* (...). *Aqui, temos de perguntar: quem é ‘diferente’ de quem?* (...). *Só se torna ‘diferente’ porque se ‘difere’ de um grupo que tem o poder de se definir como norma - a norma branca* (...). *A segunda característica é: essas diferenças construídas estão inseparavelmente ligadas a valores hierárquicos* (...). *Por fim, ambos os processos são acompanhados pelo poder: histórico, político, social e econômico.* (...). *E, nesse sentido, o racismo é a supremacia branca*”.

No cenário atual, a disseminação da interseccionalidade também suscita um debate ético-político sobre a sua apropriação e desvirtuamento, o que tem implicado o desafio adicional: resistir à sua colonização epistêmica. O poder epistêmico, exercido por comunidades interpretativas privilegiadas − entre elas, a academia −, define “*quais temas são dignos de investigação*” ^9^ (p. 183). Geralmente, esses temas apoiam o *status quo* e, por isto, não enfrentam restrições institucionais, replicando as hierarquias sociais existentes ^9^. O branqueamento da interseccionalidade é um dos maiores desafios, pois tende a descentralizar “*o papel da raça no pensamento e na práxis interseccional*” ^15^ (p. 11).

No Brasil, essas disputas epistemológicas se entrelaçam a uma tradição de negação do racismo, que historicamente interditou o debate sobre as injustiças sociais dele decorrentes. O discurso da democracia racial silenciava qualquer menção ao racismo, tornando até o termo “negro(a)” um tabu ^16^. Registros da escravidão foram apagados, impedindo a criação de um discurso fiel sobre essa tragédia fundadora de nossa sociedade. “*Por mais que o discurso pareça algo insignificante, as interdições que o atingem logo revelam sua ligação com o desejo e o poder*” ^17^ (p.10).

A produção discursiva sobre a raça começa em um racismo que legitimou o sequestro, a morte e a subjugação de africanos e seus descendentes e, mais tarde, foi reformulada pelo racismo científico, que naturalizou a inferioridade dos corpos negros. Organizado em torno de contingências históricas − modificáveis, porém continuamente atualizadas, como se observa no racismo algorítmico −, esse processo deu origem e sustenta o racismo estrutural contemporâneo ^18^.

A consolidação do mito da democracia racial foi acompanhada de aparatos jurídicos que mantiveram o s*tatus* de subcidadania do povo negro − escolhas políticas, e não condições naturais, que permitiram a perpetuação sistêmica do racismo ^19^. Os eugenistas, respaldados por diversas ciências, traziam “verdades” sobre a inferioridade dos negros, enquanto o patriarcado fazia o mesmo em relação às mulheres, criando supostas verdades com base em quem detinha o direito de enunciar o discurso.

A engrenagem de poder/saber que historicamente produziu discursos de inferiorização, desloca-se quando novas vozes ocupam os espaços de produção do conhecimento. A presença de mulheres negras na Academia tensiona o regime de verdades e abre fissuras nas formas hegemônicas de enunciar o mundo. Surge, então, a questão: que nova “vontade de verdade” pode emergir se, como afirma Foucault ^17^ (p. 17), o saber opera segundo as normas sociais? O discurso hegemônico torna-se mais rarefeito e, como observa Ynaê Santos ^19^, isto representa um “presente de grego” para a Academia, pois essas mulheres trazem memórias invisibilizadas que emergem em momentos de ruptura e transformação.

### A interseccionalidade-ferramenta

A segunda questão sobre o uso da interseccionalidade diz respeito à sua relevância nas ações voltadas à justiça social ^8^. As autoras alertam que discutir interseccionalidade não equivale a promover justiça social, pois a desigualdade resulta da interação de múltiplas categorias de poder, raramente de um único fator. Assim, no campo da saúde, a interseccionalidade funciona como ferramenta analítica para compreender o processo saúde/adoecimento. Como observa Beauvoir ^20^ ao tratar do “Sujeito Absoluto” (o homem), ainda persiste − embora em superação − a tendência dos estudos em saúde de equiparar as experiências dos homens brancos às da sociedade em geral.

O poder é dinâmico: gera respostas políticas e compõe uma complexa matriz de desigualdades. Por isso, é fundamental considerar as diferentes “categorias de poder”. Identidades como raça, classe e gênero são centrais para compreender dinâmicas sociais e enfrentar relações interseccionais. O foco, contudo, deve recair sobre os “ismos” − racismo, classismo, sexismo, etarismo e capitalismo −, raízes estruturais das desigualdades. A lente multifocal dos estudos interseccionais é decisiva para entender as opressões que atravessam sujeitos com múltiplas identidades. Como afirmam Almeida et al. ^15^ (p. 8), “*o feminismo ou a luta antirracista deve sempre ser anticapitalista, caso contrário não modifica as estruturas sociais mais profundas das desigualdades*”.

A interseccionalidade evidencia como as pessoas são afetadas por quatro domínios do poder: estrutural (saúde, habitação, trabalho, educação), cultural (ideias e representações), disciplinar (práticas de controle identitário) e interpessoal (a convergência dos demais, que molda experiências individuais). Esses domínios explicam por que certos grupos ocupam posições desiguais e por que alguns jamais alcançam a plena cidadania. As desigualdades econômicas decorrem (entre outros fatores) de práticas laborais que impõem barreiras ou privilégios segundo a identidade. O capital sempre interseccionou os corpos que produzem trabalho, já a lógica neoliberal e o discurso meritocrático transferem ao indivíduo a culpa por problemas estruturais: “*a resolução dos problemas sociais se resume à autoconfiança dos indivíduos*” ^8^ (p. 37).

É necessário um “*engajamento dialógico como estrutura orientadora para a interseccionalidade*”, pois as epistemologias e metodologias estão diretamente envolvidas no desenvolvimento ou na supressão de conhecimentos de resistência, e não são neutras. Isso reforça a ideia de ação, já que “*a experiência de fazer interseccionalidade é práxis*”, e essa práxis influencia a teorização interseccional ^9^ (p. 25).

Como teoria social crítica, a interseccionalidade se enriquece no diálogo sensível e na tensão criativa entre o saber − investigação e reflexão intelectual após a incorporação acadêmica − e o fazer − práxis e ação política originadas no ativismo social. Ela desafia o binarismo que separa teoria e prática, estudos e ativismo ^9^. Destaca a importância de pessoas historicamente privadas de direitos serem agentes desse conhecimento, bem como a necessidade de um olhar autorreflexivo sobre o que é produzido. Propõe engajamento com “*outras tradições de pensamento emancipatório*” ^9^ (p. 258) sem reproduzir hierarquias de conhecimento e poder, democratizando-os. É teoria que não se limita a explicações complexas sobre desigualdades, mas busca corrigir essas injustiças por meio da análise e ação social.

Assim, como conceito e método, nos ajudou a escrutinar algumas significativas dinâmicas políticas, econômicas e sociais das desigualdades, no que se refere ao contexto latino-americano, com destaque para o brasileiro, num recorte temporal relativo à década anterior à pandemia da COVID-19, e nos anos imediatamente posteriores. Para tanto beneficia-se de fontes de dados de instituições de reconhecida importância nas análises no contexto latino-americano, tais como Comissão Econômica para a América Latina e o Caribe (CEPAL); Instituto de Pesquisa Econômica Aplicada (IPEA); Banco Mundial; Organização das Nações Unidas Mulheres (ONU Mulheres); Escritório das Nações Unidas sobre Drogas e Crimes (UNODC); Instituto Brasileiro de Geografia e Estatística (IBGE); Organização das Nações Unidas para a Alimentação e a Agricultura (FAO); Rede Brasileira de Pesquisa em Soberania e Segurança Alimentar (PENSSAN). O estudo beneficia-se também de revisões sistemáticas publicadas em revistas científicas de reconhecida importância no campo da Saúde Coletiva.

## Condições de vida e saúde das mulheres latino-americanas - desigualdades interseccionais de negras brasileiras

As desigualdades sociais na América Latina se ancoram na continuidade de um padrão de opressão e acumulação forjado pelo colonialismo escravista e patriarcal, e reatualizado no capitalismo. Nessa ordem, o lugar de sujeitos e coletivos decorre da intersecção de camadas de opressão segundo corpo, gênero, raça, classe e origem. Mulheres latino-americanas ocupam uma posição de extrema desvantagem. As desigualdades interseccionais não são resíduos, mas pilares constitutivos do sistema, preservados pela associação de interesses políticos e econômicos desde a colonização, e mantidos na colonialidade contemporânea.

Essa tessitura histórica faz com que desvantagens se conformem, recaiam e se perpetuem sobre mulheres − aqui especialmente discutidas as afrodescendentes − com efeitos deletérios sobre trabalho, vida e saúde. A análise demográfica, econômica e epidemiológica dessas populações é dificultada pela insuficiência e má qualidade de dados, expressão da colonialidade do saber que invisibiliza arranjos supremacistas e, por vezes, nega o estatuto de humanidade a esses povos ^1^.

Um conjunto significativo de desafios enfrentado em toda a América Latina e Caribe é apontado por CEPAL no documento intitulado *Panorama Social de América Latina* ^21^, no qual há o reconhecimento da não inclusão de questões relativas a desigualdades étnico-raciais nas agendas governamentais, cujo cenário é ainda mais agravado em função da má qualidade dos registros quando estes são residualmente realizados. Em 2018, reconhece que a falta de informação estatística adequada sobre pessoas afrodescendentes é uma “dívida dos Estados”, o que tolhe o avanço de políticas de igualdade − inclusive de gênero − e que o incremento observado decorreu sobretudo da pressão de organizações negras e de agências do sistema Organização das Nações Unidas (ONU) ^22^.

Em perspectiva interseccional, o “desconhecimento” decorre de apagamentos intencionais que sustentam o projeto da colonialidade, ocultando a base moderno-capitalista fundada na racialização do Sul Global e na exploração de territórios e sujeitos subalternizados por classe, raça e gênero. Assim, as dinâmicas políticas, sociais e econômicas regionais afetam negativamente modos de vida e perfis de adoecimento de pessoas negras − sobretudo mulheres − que enfrentam barreiras históricas de acesso à educação, saúde, trabalho e representação política. Embora a última década tenha ampliado a atenção às políticas de igualdade na América Latina e no Caribe, especialmente no pós-pandemia, persistem profundas disparidades econômicas, sociais e raciais ^23^.

No campo educacional, o Panorama Social ^24^ mostra que, em 7 de 11 países com dados, a permanência escolar de 12-17 anos é menor entre afrodescendentes do que entre não afrodescendentes e não indígenas (diferenças mais evidentes no Uruguai, Equador e Venezuela; tendência também na Colômbia, Costa Rica, Bolívia e Brasil). Apesar da expansão educacional das últimas décadas e de alguma redução das desigualdades − sobretudo no Ensino Fundamental −, persistem *gaps* que agravam com a idade, favorecendo as mulheres brancas em comparação com as negras e indígenas ^24^.

No campo do trabalho e da renda, a penalidade racial persiste: trabalhadores brancos ganham, em média, 69% mais do que negros, independentemente da escolaridade. Mulheres negras concentram-se em ocupações informais e domésticas, com baixa proteção social e remuneração − 44% inferiores às dos homens brancos ^22^. Em 2023, estimativas regionais apontam que homens recebem cerca de 28% a mais do que mulheres; entre elas, as brancas ganham 30% a mais do que negras e indígenas ^24^. No Brasil, o IPEA ^25^ revela a informalidade do trabalho doméstico − majoritariamente exercido por mulheres negras −, com 2,5 milhões de diaristas em 2019 e 40% delas em regime precarizado: “*o trabalho alternado em diversos lares apresenta-se como saída* (...) *sem a exigência legal de formalização de vínculos empregatícios*” ^25^ (p. 56).

Quanto à renda, o Banco Mundial ^26^ aponta que “a *probabilidade de ser pobre é maior entre as famílias chefiadas por mulheres afrodescendentes em todos os países para os quais existem dados estatisticamente significantes*” ^26^ (p. 74). A CEPAL ^27^ indica que a renda média de mulheres afrodescendentes não supera o nível de vulnerabilidade, e que a insuficiência de renda sustenta desigualdades sociais e raciais mesmo com maior escolaridade. Mulheres indígenas têm três vezes mais probabilidade de viver em extrema pobreza do que as não indígenas ^28^. Agrega-se a essa análise a questão da gravidez na adolescência, mais frequente entre mulheres de baixa renda − majoritariamente afrodescendentes −, que reforça a exclusão educacional e perpetua a pobreza intergeracional na América Latina, conforme o relatório *Niñas y Adolescentes en América Latina y el Caribe: Deudas de Igualdad* ^29^.

O relatório *La Autonomía Económica de las Mujeres en la Recuperación Sostenible y con Igualdad* ^30^ mostra que a pandemia de COVID-19 agravou as desigualdades interseccionais, afetando sobretudo mulheres afroindígenas na América Latina e no Caribe. A crise gerou retrocesso de mais de uma década na participação feminina no mercado de trabalho. Em parceria com a Organização Internacional do Trabalho (OIT), foram identificados os setores mais vulneráveis à perda de empregos − comércio, indústria, turismo e serviço doméstico −, que concentram 56,9% do emprego feminino e 40,6% do masculino na América Latina, e 54,3% e 38,7% no Caribe. Esses setores, que empregam majoritariamente mulheres negras, mantêm altas taxas de informalidade, baixos salários e pouca qualificação ^30^.

Como a escolaridade é de grande importância em relação aos níveis de emprego e renda, impactando as condições de vida e saúde da população, no Brasil, segundo dados do IBGE, trabalhados em documento especial do Ministério da Igualdade Racial sobre mulheres negras, a taxa de analfabetismo destas é de 6,9% e de brancas é de 3,4%. Em relação ao acesso ao ensino superior completo, a desigualdade é ainda maior, sendo de 14,7% e 29% para mulheres brancas e negras, respectivamente ^31^. A colonialidade de gênero e raça ^1^ ^,^ ^2^, associada à noção de imagem de controle ^32^ contribuem para o entendimento de um fenômeno trazido no documento, ao constatar que em 2020, 33,2% das mulheres negras, ainda que tendo Ensino Superior, encontravam-se em postos de trabalho que exigem apenas Ensino Fundamental ou Médio.

A noção de imagem de controle contribui para analisar a cultura de violência que recai sobre os corpos das mulheres, especialmente das negras. Apesar da subnotificação, estudos indicam um aumento global da violência doméstica durante a pandemia de COVID-19. As restrições de mobilidade intensificaram a vulnerabilidade de mulheres já submetidas a ambientes domésticos violentos. A revisão sistemática identificou alta prevalência de violência psicológica entre aquelas em situação de violência prévia ^33^, associando-a ao estresse econômico, ao confinamento e à cultura misógina patriarcal presente nas relações de gênero.

Uma publicação recente da ONU Mulheres ^34^ denuncia a persistência inaceitável da cultura de violência de gênero, evidenciada pelos números de feminicídios em todo o mundo. Em 2023, 60% das 85 mil mulheres e meninas assassinadas foram mortas por parceiros íntimos ou outros familiares, totalizando cerca de 51.100 vítimas. O estudo faz um importante alerta: embora nas últimas duas décadas tenha aumentado o número de países que reportam dados sobre assassinatos de mulheres e meninas por parceiros íntimos ou outros familiares − alcançando 75 países em 2020 −, observou-se uma redução em 2023. O relatório constata que, apesar de os Estados-Membros terem adotado medidas para enfrentar os assassinatos relacionados ao gênero, tais ações não têm sido acompanhadas pela necessária disponibilidade e qualidade dos dados.

A matriz cis-hetero-patriarcal sustenta a objetificação e a ideia de propriedade sobre o corpo e a existência das mulheres, acentuando desigualdades quando se trata de negras, trans e travestis. O referido estudo, contudo, apresenta uma lacuna relevante ao não desagregar os dados por raça/cor nem mencionar essa ausência, tampouco incluir informações sobre identidade de gênero, adotando uma perspectiva binária e naturalizada. Além disso, utiliza o termo “mulher” de forma homogênea, desconsiderando-o como construção social e ignorando as múltiplas identidades que, nas interseções de gênero, sexualidade, raça e classe, produzem desigualdades nas vivências de violência ^34^.

No Brasil, o IBGE ^35^ indica que 18,3% das pessoas com 18 anos ou mais relataram ter sofrido violência física, psicológica ou sexual nos 12 meses anteriores, segundo a *Pesquisa Nacional de Saúde* de 2019. As mulheres foram mais vitimizadas do que os homens (19,4% e 17%, respectivamente), com maior proporção entre as mulheres pretas (21,3%) ^36^. O Fórum Brasileiro de Segurança Pública (FBSP) registrou aumento de feminicídios em 2023, totalizando 1.463 vítimas − 1,6% a mais do que em 2022, quando houve 1.440 casos ^36^.

O cenário das desigualdades étnico-raciais e de gênero no Brasil revela, na fome, uma de suas faces mais perversas. Embora o país tenha apresentado avanços recentes − retornando ao mapa da segurança alimentar, segundo a FAO ^37^, em razão das políticas sociais implantadas pelo governo do presidente Luís Inácio Lula da Silva, reeleito em 2023 −, o quadro permanece complexo. Persistem desafios significativos para reverter o agravamento das condições de vida provocado pelos dois governos liberais anteriores, que deixaram um passivo social de difícil reparação.

O relatório do *II Inquérito Nacional sobre Insegurança Alimentar no Contexto da Pandemia da COVID-19 no Brasil* (VIGISAN), de 2022, aponta a dimensão do problema, e já em sua apresentação, destaca: “*Por trás da fome, temos o flagelo sobre as crianças, as mulheres e a população negra...*”. No ano de 2022, a Insegurança Alimentar Grave aumentou em todos os domicílios, independentemente da raça/cor, porém o aumento foi maior naqueles domicílios com pessoa de referência negra (preta/parda), saindo de 8,7% para 10,2%, e nos domicílios com pessoa de referência branca, de 3,3% para 6,6%. O documento destaca então a “*permanência de prevalência de insegurança alimentar grave de maior magnitude em domicílios chefiados por pessoa negra*” ^38^ (p. 80).

A dimensão racial da fome revela-se mais preponderante do que outros marcadores sociais. O documento evidencia que, mesmo entre famílias chefiadas por mulheres negras com oito anos ou mais de escolaridade, o acesso à educação não impediu a situação de vulnerabilização: cerca de um terço desses lares vivencia insegurança alimentar moderada ou grave. Em comparação aos demais, os porcentuais são assim distribuídos: 21,3% de homens negros, 17,8% de mulheres brancas e 9,8% de homens brancos. Esse panorama da fome reproduz a já conhecida pirâmide social de base racializada brasileira, na qual o último degrau é ocupado por mulheres negras e o topo por homens brancos ^38^.

Assim, articula-se a noção de determinação social da saúde, conceito chave da aposta da saúde coletiva brasileira e da medicina social latino-americana, dado que ancorada na relação entre as dimensões econômicas, político-sociais e étnico-raciais com as diferenças nas condições de vida e saúde de populações às perspectivas da decolonialidade, ao consubstanciar histórica e materialmente as explorações e opressões submetidas ao Sul Global ^1^ ^,^ ^2^ ^,^ ^3^ ^,^ ^23^, e da interseccionalidade para discutir como camadas interpostas de opressão intensificam a vulnerabilização das condições de saúde de mulheres negras ^9^ ^,^ ^11^ ^,^ ^32^. No período de 2016 a 2022, vimos no Brasil um movimento de desmonte de políticas sociais fruto de crise política, de governo de extrema direita, e também associado à pandemia da COVID-19, tornando essas desigualdades ainda mais visíveis, sobretudo em indicadores como a mortalidade materna ^39^. Góes et al. ^40^ (p. 2507) apontam maior letalidade entre mulheres pretas (17,9%) e pardas (15,1%) em relação às brancas (13,3%), evidenciando que “*o racismo e as desigualdades raciais afetam gestantes e puérperas pretas e pardas, expondo-as a maior risco de desfecho letal associado à COVID-19*”. Os estudos *Nascer no Brasil I* e *II* confirmam a disparidade: entre 2015 e 2022, a taxa de mortalidade materna foi de 100,38 para mulheres pretas, 50,36 para pardas e 46,56 para brancas ^41^. A disparidade racial na mortalidade materna é presente também no continente latino-americano. No período de 2020 e 2021, 92,2% das mortes maternas associadas à COVID-19 na região ocorreram entre mulheres indígenas e negras ^39^.

A histórica opressão interseccional na região atua no sentido da manutenção das desvantagens em saúde que recaem sobre pessoas racializadas, em especial mulheres negras e indígenas. Esse cenário é ainda mais aprofundado quando em um contexto de crises político-econômicas e de emergências sanitárias, como a pandemia da COVID-19, conforme problematiza-se a seguir.

## Autonomia da mulher negra em cenário pandêmico

Campoalegre Septien et al. ^42^ descrevem os impactos da pandemia sobre três dimensões de autonomia, que se inter-relacionam e influenciam mutuamente, especialmente quando analisadas sob a lente da interseccionalidade entre raça, gênero e classe. Entre elas, destaca-se a autonomia econômica, entendida como a capacidade de controlar recursos, acessar o mercado de trabalho, dispor de renda própria, estabilidade no emprego e independência financeira. Essa dimensão é fortemente impactada pela divisão sexual e racial do trabalho − herança do colonialismo − que mantém as mulheres afrodescendentes em posições desvantajosas, concentrando-as em atividades informais, domésticas ou de baixa remuneração, muitas das quais foram classificadas como “essenciais” durante a pandemia.

No contexto pandêmico, essas desigualdades aprofundam, mulheres negras sofreram mais perdas de emprego, tiveram redução de renda e ficaram mais vulneráveis ao adoecimento em razão da dificuldade de cumprir as medidas de distanciamento social e confinamento. No Brasil, a pobreza incide majoritariamente sobre a população negra, limitando seu acesso à educação, aos serviços, à qualificação profissional e aos espaços de decisão ^42^. Dados do IBGE ^43^ reforçam essa assimetria: 37,7% das pessoas pretas e pardas vivem abaixo da linha da pobreza, praticamente o dobro da proporção observada entre pessoas brancas (18,6%).

A autonomia física refere-se à capacidade de decidir livremente sobre a própria sexualidade, reprodução e o direito a uma vida livre de violências. Envolve o acesso à saúde, a serviços de saúde sexual e reprodutiva, e o exercício pleno do direito sobre o próprio corpo. Para Campoalegre Septien et al. ^42^, a violência de gênero − presente tanto nos espaços públicos quanto privados − compromete gravemente essa autonomia, especialmente entre as mulheres afrodescendentes. O feminicídio constitui sua expressão extrema e as mulheres negras figuram como as principais vítimas: no Brasil, representaram 73% dos casos registrados.

No primeiro semestre da pandemia, em 12 estados brasileiros houve aumento de 3,8% nas chamadas à Polícia Militar por violência doméstica. Em Bogotá, Colômbia, o crescimento foi de 187%, concentrado em casos de violência psicológica. Gutiérrez et al. ^44^ apontam que a intensificação da violência intrafamiliar decorreu da cultura machista e patriarcal, agravada pelo isolamento social. Esse cenário, contudo, ultrapassa o contexto pandêmico: pessoas negras seguem como principais vítimas de homicídios − 76% entre homens e 66% entre mulheres ^45^. Em 2022, “*mulheres negras tiveram 1,7 vez mais chances de serem vítimas de homicídio*” ^45^ (p. 41), revelando a persistência das desigualdades raciais na violência letal.

Já a autonomia decisória é fundamental para garantir às mulheres participação ativa nas escolhas que impactam suas vidas e comunidades, constituindo a dimensão central da cidadania e da democracia. No entanto, desigualdades estruturais e discriminações de gênero e raça ainda restringem esse exercício, sobretudo entre as mulheres negras. Pinheiro ^46^ mostra que sua sub-representação nos cargos diretivos do Executivo Federal compromete a justiça social e a qualidade democrática. A predominância de homens brancos nos espaços de poder perpetua vieses na gestão pública, enquanto cotas, ações afirmativas e a divisão igualitária do trabalho doméstico e de cuidados são estratégias essenciais de transformação estrutural.

Tokarski et al. ^47^ lembram que as mulheres foram historicamente mais restritas ao espaço doméstico ou sobrecarregadas pela dupla jornada. Sua participação no mercado de trabalho permanece inferior à masculina, marcada por maiores taxas de desemprego e menores salários. A intersecção entre gênero e raça agrava essas desigualdades: mulheres negras são o grupo mais marginalizado. Dados do Ministério do Trabalho e Emprego e do Ministério das Mulheres ^48^ mostram que, no Brasil, elas ganham em média 19,4% menos do que os homens; entre as negras, o rendimento corresponde a 68% da média nacional, já os homens não negros recebem 27,9% acima. Nesse cenário, Campoalegre Septien et al. ^42^ apontam a criminalização de lideranças negras como forma de feminicídio simbólico − mecanismo que silencia vozes e limita a atuação política no Brasil e na Colômbia.

## A agência de mulheres racializadas - movimentos de mulheres negras e indígenas

Em oposição à memória oficial hegemônica, narrada pelos grupos dominantes, emergem socialmente lembranças e narrativas históricas do povo negro afrodiaspórico, marginalizado e silenciado. Essas memórias, mantidas vivas por saberes africanos ancestrais, são preservadas por meio de conversas familiares, redes de amizade, manifestações culturais e, especialmente, pela tradição oral passada de geração em geração. Mais do que um registro do passado, essa memória é uma força ativa no presente, moldando como entendemos e interagimos com o mundo. Trata-se de um ato de resistência contra as diferentes formas de colonialidade e de preservação de perspectivas alternativas, como ressalta Leda Martins ^49^.

As resistências às hierarquias raciais e de gênero impulsionaram o feminismo negro, como destaca Hooks ^50^. Ao racializar o feminismo, ela evidencia que o movimento branco hegemônico relegava as mulheres negras à subalternidade, mantendo o “paternalismo neocolonial” e a supremacia branca, mesmo sob o discurso antissexista. Diante da impossibilidade de uma sororidade genuína, o feminismo negro criou sua própria base de solidariedade entre as mulheres vistas como “outras”. Ao criticar, nos anos 1970, a falta de interseção entre raça e gênero, Hooks ^50^ foi acusada de “traição” ao movimento, mas expôs que a ética do cuidado raramente se estende a grupos fora da própria identificação racial ou étnica. Também ressaltou a centralidade da classe, defendendo que só a união entre todas as mulheres pode enfrentar o patriarcado.

Chandra Mohanty ^51^ critica o feminismo ocidental por reproduzir uma lógica colonizadora ao falar em nome das mulheres do Sul Global, transformando a “Mulher do Terceiro Mundo” em categoria analítica criada pelo olhar eurocêntrico. As metodologias e discursos do norte global, ao se proclamarem universais, ignoram contextos históricos e políticos específicos, perpetuando a colonialidade do saber. Mohanty propõe desconstruir esse privilégio epistêmico e situar toda produção acadêmica como prática política e ideológica dentro das hierarquias globais que a sustentam. Embora reconheça que a opressão feminina seja um fenômeno mundial, adverte que tratá-la de forma homogênea impede avanços teóricos e políticos. Defende, assim, um feminismo decolonial capaz de enfrentar racismo, patriarcado e imperialismo com base nas realidades locais das mulheres subalternizadas.

Essa crítica a uma perspectiva eurocêntrica do feminismo anglo-saxão é endossada por Santos Madrigal ^52^, que a compreende como um paradigma universalista que nem sempre dialoga com as outras experiências, como as do Sul Global, por exemplo. Aponta tratar-se de um paradigma influenciado pelo capitalismo industrial, que invisibiliza outras formas de organização social. A própria ideia de patriarcado, como um sistema estrutural de dominação masculina, ignora a diversidade de lugares sociais; cita Oyéronké Oyewùmi, para quem a imposição de gênero como estrutura binária (entre masculino e feminino) e hierárquica serviu para estruturar relações de dominação dentro do sistema colonial. É, ainda, um conceito de mulher que “*folcloriza ou inferioriza as mulheres negras, indígenas, campesinas, imigrantes, lésbicas*” ^52^ (p. 2).

No documentário Ôrí, Beatriz Nascimento destaca a potência da coalizão e do aquilombamento: “*É preciso a imagem para recuperar a identidade. É necessário tornar-se visível, pois o rosto de um é o reflexo do outro, o corpo de um é o reflexo do outro, e cada um é o reflexo de todos os corpos*” ^53^. Essa perspectiva dialoga com o que Henning ^7^ chama de “*táticas de resistência, questionamento e desconstrução da desigualdade, sobretudo por meio de diferentes formas de agência interseccional*” ^7^ (p. 117). Como observa Borret ^4^ (p. 3971), “*as ferramentas produzidas pelo povo negro como estratégia de resistência e forma de manter alguma conexão ancestral foram (e são) duramente perseguidas: quilombos, samba, capoeira, jongo, religiões de matriz africana*”.

Essas expressões de resistência coletiva − fundadas em memória, afeto e solidariedade − sinalizam caminhos de reexistência que anunciam possibilidades de transformação social em perspectiva decolonial e interseccional.

## Considerações finais - em busca de políticas reparadoras para mulheres afro-latino-americanas e caribenhas

Como pode ser visto, as desigualdades étnico-raciais e de gênero em saúde na América Latina são graves e têm despertado cada vez mais atenção por um conjunto ampliado de agentes sociais - acadêmicos, governamentais e da *advocacy social*, no sentido de enfrentamento e mitigação de inúmeras faces das iniquidades. Ações concertadas em nível do continente latino-americano precisam entrar com mais potência nas agendas de países e nas agências multilaterais de cooperação.

Um conjunto significativo de *policy briefs* submetidos à chamada para contribuições de *think tanks* na Reunião do G20 Brasil, especialmente no âmbito da *Task Force* “*Fighting Race and Ethnic Discrimination and Inequalities*”, apresenta propostas consistentes e relevantes para os formuladores de políticas dos países latino-americanos e caribenhos. Grande parte dessas contribuições enfatiza a necessidade de implantação de políticas de reparação voltadas a mulheres afro-latino-americanas e indígenas, compreendidas como estratégia de desenvolvimento social com equidade. Tais políticas produzem efeitos ampliados sobre a sociedade, pois visam a melhorar as condições de vida das famílias e reconhecem o papel social das mulheres historicamente vulnerabilizadas e subalternizadas ^54^.

A urgência imposta pelo contexto aqui debatido consubstancia-nos em propor a priorização da inserção destacada desse grupo social, em um significativo conjunto sinérgico de políticas públicas, numa perspectiva intersetorial, estrategicamente conformadas. A formulação de políticas voltadas para essas mulheres parte do reconhecimento de seu impacto social, considerando o papel central que as mulheres racializadas ocupam no tecido social, estando estas na base da pirâmide socioeconômica. Experiências exitosas, como o Programa Bolsa Família no Brasil, ilustram esse potencial, ao apresentarem efeitos significativos na redução das desigualdades de renda, na melhoria da saúde materno-infantil e na diminuição da morbimortalidade por violências, entre outros resultados positivos ^55^.

Marques et al. ^56^ destacam a importância da representatividade política da população negra em espaços decisórios. Para os autores, o aumento dessa participação requer medidas estruturantes, como a criação de reservas orçamentárias para programas e políticas antirracistas, cotas para candidaturas negras, ocupação de cargos ministeriais compatíveis com a proporcionalidade da população negra e a garantia de equidade de gênero nesses cargos.

Nesse horizonte, é preciso reconhecer que as lutas dessas mulheres ultrapassam o campo das políticas institucionais: são também expressões de saber, memória e resistência histórica. No entrelaçamento entre corpo, memória e ancestralidade, as mulheres negras latino-americanas e caribenhas seguem produzindo outras epistemologias, outras formas de mundo e de existência − marcas de um projeto de vida que insiste em existir, resistir e transformar; afinal, como lembra Angela Davis ^57^, “*quando a mulher negra se movimenta, toda a estrutura da sociedade se movimenta com ela*”.

## Data Availability

As fontes de informação utilizadas no estudo estão indicadas no corpo do artigo.
